# Adulthood as a social construct: Critical discourse analysis of transition preparation consultations for youth with chronic conditions

**DOI:** 10.1016/j.hctj.2026.100145

**Published:** 2026-06-16

**Authors:** Maxime Morsa, Clémentine Boudroit, Agnes Dumas, Enora Le Roux, Paul Jacquin, Hélène Mellerio

**Affiliations:** a Psychology Department, Adaptation, Resilience & Change (ARCh) Research Unit, University of Liège, Liège 4000, Belgium; b Laboratoire Éducations et Promotion de la Santé (LEPS UR3412), Université Sorbonne Paris Nord, Bobigny, France; c Université Paris Cité, ECEVE, UMR 1123, Inserm, 10 avenue de Verdun, 7, Paris 5010, France; d AD’venir, Adolescent Medicine Unit, Robert Debré Hospital, Assistance Publique-Hôpitaux de Paris, 48 bd Sérurier, Paris 75019, France

**Keywords:** Critical discourse analysis, Qualitative research, Illness identity, Transition, Preparation consultation

## Abstract

**Background:**

Little is known about how interventions designed to empower youth with chronic conditions operate in natural clinical contexts during the transition from pediatric to adult care. Such interventions may contribute to social learning through discourse about the future, illness, and adolescence, thereby shaping youths’ understandings of identity and empowerment. This study examined how meanings of transition are discursively constructed during Transition Preparation Consultations (TPCs), a structured intervention delivered within the AD’venir transition unit in France for youth with diverse chronic conditions.

**Methods:**

We conducted a critical discourse analysis of 26 TPCs, focusing on the discursive resources and positioning practices through which youth and physicians constructed meanings of transition.

**Results:**

TPCs framed transition as a psychological and social process, encouraging youth to develop experiential understandings of illness and identity. Consultations primarily focused on self-management, living with a chronic condition in adulthood, and health maintenance, while discussions were frequently anchored in bodily changes associated with adolescence. Although physicians often adopted pedagogical and dialogic forms of communication, interactions remained largely structured by prescriptive discourses regarding responsibility, self-management, and adult health behaviors.

**Conclusions:**

Transition programs function not only as organizational interventions but also as discursive spaces in which meanings of illness, responsibility, autonomy, and adulthood are actively produced. Recognizing these processes may support critical reflection and the ongoing refinement of transition practices.

## 1 Background

The experience of transitioning to adulthood for youth with chronic conditions (CC) is considered challenging due to a variety of factors. These include the shift from pediatric to adult care, the empowerment required to manage the condition, and the multiple physical, psychological, and social changes experienced by the youth with CC.^1^, ^2^ Risks of poor well-being, non-adherence to treatment, and interruption in medical follow-up increase during this period.^3^ Healthcare transition involves supporting and preparing youth with CC not only to move from pediatric to adult care but also to become an adult while managing the constraints of a CC. This process begins in early adolescence and continues into young adulthood with the aim of promoting their health, empowerment, and well-being.^4^

Currently, atheoretical approaches to transition persist in the literature, highlighting the need for studies grounded in robust theoretical frameworks.^5^ From this perspective, we propose to examine this transition to become an adult with CC as a social learning phenomenon, drawing on work in sociocultural developmental psychology.^6^ Indeed, healthcare transition engages youth with CC in their personal development, shaping their perceptions of adulthood with a chronic condition and its possibilities. Socio-cultural approaches of human development emphasize that transitional periods function as learning processes during which individuals acquire new knowledge, reconfigure their social roles, and engage in meaning-making work about their experiences of change.^7^ Youth with CC thus gradually learn to become adults through experiences (such as self-managing their care, scheduling medical appointments, and attending consultations without their parents) and through a process of socialization that takes place during interactions with the healthcare system, involving social discourses surrounding these experiences (including what an adult with a chronic condition can or cannot do, what is expected of them, what they should be cautious about, etc.). We draw on the work of Erving Goffman ^8^ to argue that social discourse contributes to the formation of a “frame of experience,” referring to the set of interpretive frameworks that young people with CC use to make sense of a situation and understand their lived experience. Discourses can be understood as structured sets of statements that shape both the way objects are perceived and the positions individuals may occupy in relation to them.^9^ These discourses are conveyed through healthcare transition interventions as mechanisms for creating shared meanings.^10^ From this perspective, learning is not only cognitive but also social and cultural, considering the interactive processes that help individuals make sense of practices and influence their personal perceptions.^11^ In the same way, learning in not only explicit and functional (self-management) but also implicit and identity-related through the construction of meaning about oneself and one’s position within social interaction.

Current research primarily documents the effects of formalized learning from healthcare transition interventions on the acquisition of knowledge,^12^ without producing insights into how these interventions inform youth with CC about themselves and their perceptions of adulthood. However, social sciences have shown that youth with CC seek care that acknowledges their identity as individuals, not solely as patients.^13^ Indeed, process of health identity is central during adolescence, while it unfolds under the threat of social stigmatization.^14^

Therefore, we need to study within real-world settings how healthcare transition facilitates the youth with CC learning of becoming an adult with a chronic condition through discourse and practices. On one hand, we know that discourse that is perceived as too prescriptive and focused on the role of being a patient can have negative effects.^15^ On the other hand, health psychology research has extensively demonstrated how the discourse used in care interactions shapes the experience of illness and care for youth with CC.^16^

In this perspective, we studied the Transition Preparation Consultation (TPC) implemented at the AD’venir transition unit (Robert Debré Pediatric University Hospital, Paris, France), using a developmentally appropriate healthcare approach.^17^ TPC is a specialized medical appointment for youth with CC, lasting almost 1 h and conducted by a physician trained in adolescent medicine, who is independent from the team that usually provides their healthcare. The purpose of TPC is to assess youth with CC needs and help healthcare providers tailor their preparation for the transition to adult care. A preliminary study detailed the general framework of TPC and demonstrated their feasibility.^18^ We now aim to study TPC as a social learning situation and to understand how TPC produces discourse about the transition towards adulthood. While healthcare transitions are characterized by the goal of preparing youth with CC for self-management and empowerment, we explore how the discursive resources mobilized by the interlocutors in the TPC process create a (new) social reality of self-management and empowerment and the experience of becoming an adult with a chronic illness.

## 2 Method

a. Type of Study

We conducted a critical discourse analysis (CDA) of interactions occurring during TPC. CDA focuses on discursive resources as they inform the identity and positioning of actors.^19^ Given that pediatric transition is now widely recognized as a youth empowerment process, CDA appeared particularly relevant for examining how power positions are constructed and distributed through the discourses produced within TPC. This approach aims to show how language (choice of words, grammatical constructions, rhetorical strategies, etc.) is involved in constructing shaping the social and psychological realities of transitioning to adulthood with special health care needs. The analysis thus focuses on the effects of discourse and the interpretive repertoires mobilized to make sense of experiences.^20^, ^21^ The analysis is referred to as “critical” particularly in reference to the work of Michel Foucault, in that it focuses on the role of discourse in processes of power and legitimation, as well as on the social practices through which institutions (such as hospitals) contribute to the construction of identities through language and its uses.^22^

As part of this study, we adopted a constructionist epistemology,^23^ considering that people's experiences and cognitions are shaped by the social and environmental context in which they are expressed. Within our study, we postulate that TPC represents a specific interactional context in which discourse shapes the reality of adulthood, health, illness, identity, and social roles. The research project aligns also with an intervention policy, following the discourse study approach described by Stokoe et al.,^24^ involving a partnership between the healthcare team (*AD’venir* transition unit) and the research teams, with the aim of improving practices that promote the health, well-being, and empowerment of youth with CC. Therefore, the care team (PJ and HM) engages in a process of self-reflexivity aimed at improving its ability to provide TPCs within a framework of developmentally appropriate healthcare.

We engaged in a reflexive process guided by the COREQ checklist.^25^ The research team that led the analytical process (CB and MM) aligns itself with an empowerment-oriented approach to health research, viewing individual agency as the ultimate goal of any non-pharmacological intervention in care. Their disciplinary background is in social health psychology.^26^ They were not involved in the design or implementation of the TPCs. Rather, they were invited by the team that initiated the TPCs (AD, EL, PJ, and HM) to conduct an evaluative study of the TPCs recorded by this team. The analyses conducted by the two researchers were presented to the entire team during three working sessions in order to strengthen their relevance and credibility in light of the clinical care context. Although the analysts were familiar with the broader healthcare and sociocultural context in which the TPCs were implemented, they were external to the clinical setting and not involved in patient management, which limited potential role-based bias.

b. Data collection and sample

The study focuses on 26 TPCs conducted between May 2017 and September 2018, at the launch of the *AD’venir* transition unit, which represent all the interviews carried out during the study period set by the healthcare team to test and document their consultation model. The TPC was introduced by the referring healthcare professional (the clinician who routinely provides care to adolescents) to all young people aged 15–25 years with a chronic condition or disability receiving care at Robert Debré Hospital and expected to transition to adult healthcare within the subsequent two years. For each TPC in the study, it was the first time the physicians met the patients. The 26 TPCs were audio-recorded with the participants' consent and transcribed into verbatim records. All patients who were approached agreed to participate in the study.

The TPC was open to all youth with CC (ages 14–25) at Robert Debré Hospital, who were supposed to be transferred to adult healthcare within the following 2 years.

During the TPC, a physician of *AD’venir* transition unit meets with the youth with CC alone to explore their perceived experience and views of the chronic condition during an unstructured interview. The consultation also includes a structured interview using the HEADSS checklist^27^; completion of the Treatment Burden Questionnaire (TBQ) ^28^ and the Good2Go questionnaire^29^ ; and a physical examination. Details on TPC process are detailed elsewhere.^18^

In total, 30 participants were involved in the study material: 26 youth with CC (11 girls and 15 boys) aged 14–23 years (mean age = 17.3 years) and 4 physicians who conducted the TPCs (See Table 1 and Table 2). Given the predominance of one physician in the analyzed TPCs, we did not specifically investigate inter-individual differences among physicians, except in cases where a significant phenomenon was identified during the analysis.

**Table 1:** youth with chronic conditions sample description.

**Name**^a^	**Age** (years old)	**Sex**	**Chronic conditions**	**Details**	**Duration of TPC** (in minutes)	**Presence of family members at the end of the TPC**
Abdoul	17	Male	Allergic asthma	Disease responsible for episodes of dyspnea, need for daily drug intake, risk of critical event	69	Yes (mother)
Abdel	23	Male	Anorectal malformation Uropathy	Rare digestive and urinary malformation, multi-operated during the first months of life, responsible for urinary and sexual problems, long-term need for multi-daily urinary catheterization	67	No
Antoine	18	Male	Atypical and syndromic microvilli atresia	Rare disease responsible for chronic diarrhea and abdominal pain, need for parenteral feeding by catheter	80	Yes (mother)
Jeanine	19	Female	Chronic intestinal pseudo-obstruction	Rare disease responsible for abdominal pain and digestive obstructions episodes, sometimes associated to a mild mental impairment, need for a digestive stoma	63	Yes (mother and father)
Zineb	18	Female	Craniopharyngioma	Rare benign cerebral tumor, post-operative sequelae such as endocrine disorders and small size, need for long-term daily drug intake	77	Yes (brother)
Ilana	18	Female	Crohn's; Hemorrhagic rectocolitis	Rare pulmonary disease responsible for chronic diarrhea, abdominal pain, fecal urgency, and pubertal delay, need for regular injections at the hospital and regimen, risk of long-term complications;	75	Yes (mother)
Juliette	18	Female	Crohn's; Hemorrhagic rectocolitis		80	No
Kensia	17	Female	Crohn's; Hemorrhagic rectocolitis		50	No
Mounia	16	Female	Crohn's; Hemorrhagic rectocolitis		45	Yes (mother)
Ahlem	18	Female	Crohn's; Hemorrhagic rectocolitis Spondylarthrite	Rare pulmonary disease responsible for chronic diarrhea, abdominal pain, fecal urgency, and pubertal delay, need for regular injections at the hospital and regimen, risk of long-term complications; Rare disease responsible for chronic back pain and restricted mobility, need for daily drug intake, risk of long-term complications	73	No
Edouard	18	Male	Cystic Fibrosis	Rare pulmonary disease responsible for respiratory difficulties, recurrent infections, digestive disorders and pubertal delay, need for multi-daily drug intake, risk of critical event and long-term complications, premature mortality	61	No
Grégoire	18	Male	Cystic Fibrosis		40	No
Medhi	15	Male	Cystic Fibrosis		80	No
Nicolas	18	Male	Cystic Fibrosis		52	No
Nolwenn	17	Male	Cystic Fibrosis		47	No
Ronan	15	Male	Hirschsprung's disease	Rare digestive malformation, operated during the first months of life, long-term digestive disorders	55	Yes (mother and father)
Mohamed	14	Male	Laparoschisis and short small intestine Chronic diarrhea	Rare abdominal malformation, multi-operated during the first hours and months of life, responsible for chronic digestive problems like abdominal pain and diarrhea	63	Yes (father)
Léa	18	Female	Nephrotic syndrome	Rare renal disease responsible for episodes of renal dysregulation with need for daily drug intake, long-term risk of dialysis	52	No
Manil	16	Male	Spondyloarthropathy	Rare articular disease responsible for chronic back pain and restricted mobility, need for daily drug intake	76	Yes (mother)
Enora	17	Female	Type 1 diabetesCeliac disease	Endocrine disease responsible for glycemic dysregulation, need of regimen and multi-daily insulin injections and glycemic controls, risk of critical event and long-term complications; Intestinal disease responsible for digestive disorders, need for strict regimen	56	Yes (mother)
Ibtissem	16	Female	Type 1 diabetes	Endocrine disease responsible for glycemic dysregulation, need of regimen and multi-daily insulin injections and glycemic controls, risk of critical event and long-term complications	80	No
Ilyes	17	Male	Type 1 diabetes		54	No
Sofiane	17	Male	Type 1 diabetes		95	Yes (mother)
Bintou	17	Female	Unlabeled connectivitis	Rare articular disease responsible for chronic joint pain and restricted mobility, need for daily drug intake, risk of comorbidities and long-term complications	62	Yes (mother)
Lucas	18	Male	VACTERL syndrome	Rare set of malformations, multi-operated during the first years of life, responsible for urinary and sexual dysfunction, digestive disorders and sometimes a mild mental impairment	90	Yes (mother)
Olivier	17	Male	VACTERL syndrome		108	Yes (mother)

a All names mentioned are pseudonyms.

**Table 2:** Physicians sample description.

**Physician**	**Number of TPC conducted within the sample**	**Gender**	**Age (years old)**	**Number of years of experience in adolescent medicine**
1	14	Woman	40	2
2	6	Woman	27	1
3	4	Man	60	30
4	2	Woman	40	10

c. Analysis Framework

We followed the qualitative critical discourse analysis steps proposed by Willig ^30^ as follows: identifying discursive constructions (*what is being talked about?*), mapping the discourses (*how is it being talked about?*), focusing on the action orientation of discourse (*what is the discursive context producing the discourse?*), identifying the positioning of actors (*what roles are embodied by the interlocutors during the interactions?*), focusing on practice (*what practices are enabled by the discourse?*), and focusing on subjectivity (*what subjective realities emerge in the discourse?*). The analysis proceeded iteratively rather than linearly, moving back and forth between the six analytic steps described by Willig.^30^ Transcripts were read multiple times to ensure familiarization with the data. Initial coding focused on identifying discursive constructions and interpretative repertoires. Codes were generated inductively from the data and progressively refined into broader discursive patterns through constant comparison. Analytical memos were written throughout the process to document interpretative decisions and reflexive considerations. The developing analysis was discussed regularly between CB and MM in order to enhance analytical rigor and coherence. The data analysis was conducted using ATLAS.ti to support systematic coding and data management.

d. Ethics

All participants provided informed consent for participation. The research did not change the care that is usually provided. The TPCs remained strictly confidential, and the data were pseudonomized. The ethics committee of Robert Debré Hospital approved the study protocol (No.2016/268).

## 3 Results

First, we structured the presentation of the themes developed during the analysis to account for (1) *what is said*, (2) *how it is said*, and (3) *what social reality emerges from the discourse*. Next, we analyzed the discursive context of TPCs. Finally, we analyzed the positioning of the interlocutors within the discursive context of the TPCs.

a. Discursive Content: what is said?

We developed 7 themes that structure the TPC, with their prominence in the corpus following this descending order: self-management of the chronic condition, future as an adult with a chronic condition, health practices associated with adolescence, support from the social environment, bodily sensations and changes, everyday life activities, and self-development with a chronic condition (Table 3).

**Table 3:** Occurrence of themes and sub-themes.

**Themes / Sub-Themes**	**Occurences**
**Self-management of the chronic condition**	**321**
Knowledge and description of the illness and care	118
Decision making and agency	112
Constraints of the illness	51
Illness history (diagnosis)	40
**Future as an adult with a chronic condition**	**297**
Projection in adult services	124
The question of "Becoming an adult"	75
Available resources for transition	54
Professional aspirations	44
**Health practices associated with adolescence**	**254**
Physical Activity	57
Affective/Sexual life	49
Substance use	38
Screen	31
Food	31
Sleep	29
Psychological and/or physical state	19
**Quality of support from the social environment**	**191**
Friend support	43
Social life	39
Family support	39
Conflicts	7
**Bodily sensations and changes**	**162**
Pain	53
Puberty/Growth	43
Self-image	36
Fatigue	30
**Everyday life activities**	**98**
School/Education	58
Video games	13
Other recreational activities	11
Work	10
Arts	6
**Self-development with a chronic condition**	**22**
Self-concept	12
Self-confidence	6
Gaze from the others	4

i. The role of self-management

Nearly the half of TPCs content focus on discussions about the self-management of chronic condition and the future as an adult living with a chronic condition. This aligns with the inherent nature of TPCs as medical consultations dedicated to the transition to adulthood. Although biomedical concerns are present, the young person's condition is not approached strictly from a biomedical perspective but rather as a phenomenon that the young person can master, control, or at least learn to manage progressively during the transition to adulthood. In this sense, TPCs prepare the youth to adopt an agentic perspective on disease management through discussions about the knowledge they have acquired about their condition and treatments, their capacity to make health-promoting decisions, and their adherence to treatment. The chronic condition is also discussed in terms of its impact on daily life, the perceived constraints, and ways to cope with them. Thus, the discourse frames the chronic condition as an experience lived by the youth, emphasizing its subjective dimension.

“Physician: I wanted to ask you, ‘If you have to explain today to someone who doesn't know at all what you have, what illness you have, how do you go about it, what do you say?’

Edouard: Well, I would tell them… that it's a lung disease, that mainly affects my lungs and that I have trouble…

Physician: Yes…

Edouard: …I have… I have trouble breathing at times…

Physician: Hmm…

Edouard: …that I get congested and all, that my lungs are more sensitive…

Physician: Yes…

Edouard: …and… then often, well, I say, compared to someone well-known like Grégory Lemarchal [Famous French singer who died from cystic fibrosis], I say ‘I have the same illness as him’ and that's it.

H. M.: Yes, does it help you to have… a reference…?

Edouard: Yes !”

(Edouard TPC, cystic fibrosis, 18 y.o.)

TPCs go beyond the strict framework of chronic condition to encompass health in its positive (promotion of healthy behaviors) and holistic (biopsychosocial) dimensions. Self-care is thus considered from a broader perspective than just chronic condition management, by exploring and educating about health practices related to physical activity, sleep, emotional and sexual life, addictions/substance use (tobacco, alcohol, drugs), and nutrition.

“Physician: And alcohol at parties?

Ilana: Yes.

Physician: Have you ever drunk any?

Ilana: Yes, um.

Physician: Just at parties…

Ilana: Yes, but recently, uh… I went to a party, it ended pretty badly, I ended up in the hospital… but, uh… I had really drunk a lot, but actually I think I was testing my limits! (laughs.), I was testing my limits and, uh… that's it! I drank way too much vodka.

Physician: Was that the party where you drank the most?

Ilana: Yes, that's it, it was really the party where I drank the most and I didn't want my parents to find out.”

(Ilana TPC, crohn's and hemorrhagic rectocolitis, 18 y.o.)

ii. The Good2Go Questionnaire as a Discussion Tool

**Fig. 1:**
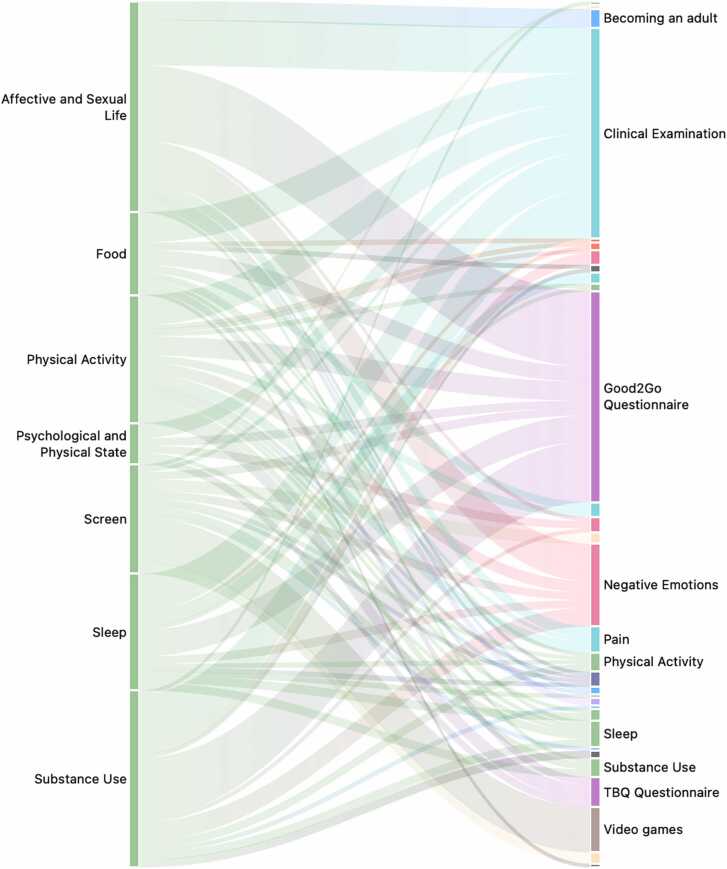
Co-occurrence analysis of health topics (Sankey Diagram).

The analysis of theme co-occurrences reveals that physicians take advantage of the opportunity presented by filling out the transition readiness Good2Go questionnaire to explore youth health behaviors more deeply, with a focus on topics such as risky behaviors. A Sankey diagram was realized with Atlas.ti software to describe this phenomenon (Fig. 1). The Sankey diagram illustrates the co-occurrence relationships among the different themes developed during the analysis, showing how they are interconnected and the strength of their links through the thickness of the flows. The diagram shows that discourse on health-related topics such as affective and sexual life, substance use, and sleep is primarily produced during the administration of the Good2Go questionnaire, whereas the other TPC session dedicated to overall health corresponds to the clinical examination. Thus, physicians go beyond the scope of the standardized tool to steer the TPC discussions towards themes specific to adolescent medicine.

While the transition to adult healthcare comprises a significant part of the discourse, TPCs are not limited to this dimension. Youth are also encouraged to articulate their own understanding of "becoming an adult" and to express their aspirations for the future. In this regard, youth often struggle to find meaning in a phase they have not yet experienced and only know through adult discourse, which often emphasizes responsibility as a constraint.

“Physician: How do you see your transition to the adult ward? How is it going to happen, do you already know some things?

Zineb: I don't really know, but I tell myself that maybe I should be more independent.”

(Zineb TPC, Craniopharyngioma, 18 y.o.)

iii. Discourse on the Body as an Experience

The transition within TPCs involves a process of constructing meaning based on the body as a site of sensations and observation of changes occurring during adolescence. Analyses show that the clinical examination during the TPC is the moment when discourse about the body emerges. This discourse establishes the social reality of the body as an experience, marking the youth identity through the exploration of sensations (pain, fatigue), observed changes (growth, puberty), and associated cognitions (self-image) and adulthood possibilities (having a child, having a job). The uniqueness of the youth, as an individual in transition within their social environment, is thus more significantly constructed through discursive resources related to the corporeal rather than strictly cognitive identity, which is a minor theme in the overall corpus.

Physician: In your daily life, is this fatigue difficult for you?

Kensia: Yes. Because… well, for example, in my mind I can feel like, okay, I’m in good shape!

Physician: Yes!

Kensia: But my body doesn’t follow, actually. (smiles)

Physician: Yes…

Kensia: Because my body is tired… maybe I also know that (smiles) I don’t know! I mean… I’m sure my body is… I don’t know, sometimes I find it strange… it doesn’t follow me.

Physician: It doesn’t follow. Alright… and what do you do in those situations? Do you push yourself? Do you try to do like everyone else, or do you tell yourself it’s not possible and then you rest? Do you take a short break first? What’s your strategy to try to…

Kensia: Sometimes I push myself, right!? Sometimes I push myself… sometimes also, well, sometimes… I can’t push myself! Because I don’t actually have the strength. I know that in my head, yes, but I don’t have the strength, I can’t find the strength to… to, well, do it.

(Kensia TPC, Crohn's and hemorrhagic rectocolitis, 17 y.o.)

^The analyses further show that perceived dissonance between representations (of self or chronic condition) and lived experience shifts the focus of the interview towards a predominance of themes related to the body or emotions. Six youth find themselves in this situation (Alhem, Kensia, Léa, Lucas, Manil, Olivier). For example, Kensia expresses a perceived gap between what she wants (*to be like others*) and what she experiences or can do (*fatigue, pain*); Manil mentions the hiatus between his perceptions ("*the illness is just a grain of sand*", the future without mention of the illness) and the "objective" reality presented to him (*treatments, medical appointments*); Léa talks about her contrasting feelings related to alternating phases of remission (*hope of recovery*) and relapse (*incomprehension*); Lucas expresses his sexual concerns while his condition (VACTERL syndrome) limits his activities, which he considers natural for his age. This dissonance generates a discourse on *being sick*, initiating the construction of meanings about what it means to be ill within the realms of young person perceptions and practices. This process engages primarily youth who express fatigue with their chronic condition, a sense of discomfort related to this social role, and who sometimes struggle to envision a future they perceive as positive.^

b. Discursive Context: How it Said?

**Fig. 2:**
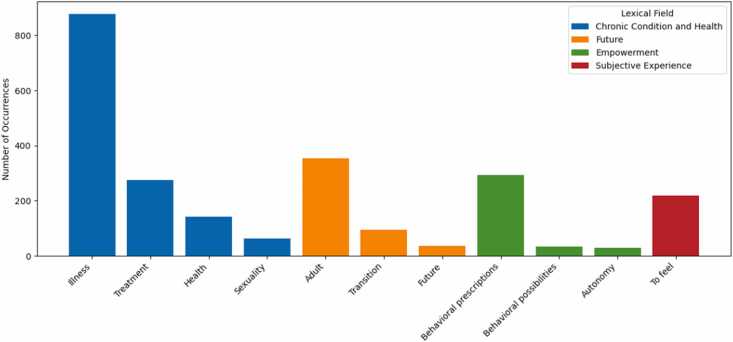
Lexical Occurrences.

^The analysis also identified several linguistic practices used by physicians that constitute significant patterns of TPCs. Firstly, the logical modalities used by physicians are divided between “deontic modality” (referring to concepts of obligation, prohibition, and permission in moral terms) and “alethic modality” (relating to expressions of possibility, impossibility, or contingency), with a significant predominance of the deontic modality. Indeed, the expressions *“you must / you should / you need”* appears 293 times throughout the entire corpus compared to only 22 occurrences of the expression *“you can / you could.”* Moreover, the expressions *“you must / you should / you need”* is primarily associated with themes of the future (122 co-occurrences), chronic condition (46 co-occurrences), and overall health (38 co-occurrences). Here are some examples:^

^The analysis of lexical occurrences identified four main lexical fields structuring the discursive context of TPCs: chronic condition and health (878 occurrences of the word "illness," 275 occurrences of the word "treatment," 142 occurrences of the word "health," 63 occurrences of the word "sexuality"), the future (354 occurrences of the word "adult," 96 occurrences of the word "transition," 37 occurrences of the word "future"), empowerment (293 occurrences related to behavioral prescriptions, 34 occurrences related to behavioral possibilities, 30 occurrences of the word "autonomy"), and subjective experience (219 occurrences of the verb "to feel"). Thus, TPCs are structured within a social reality of the transition to adulthood focusing on chronic condition management (^ Fig. 2 ^).^

“Okay, the precautions for taking the medications: you **need** to take them at specific times, before or after meals, not just any way…”

“So if you need contraception, you **should talk** to the people at the hospital…” (Bintou TPC, unlabeled connectivitis, 17 y.o.)

“You **should** start exercising again next year then! (smile)”

“Well, you should check with the Physician, you **must** talk about it, okay!?”

(Ilana TPC, crohn's and hemorrhagic rectocolitis, 18 y.o.)

^While deontic modality is framed within a perspective of youth responsibility, positioning autonomy associated with adulthood as necessary practices for effective functioning and awareness of the obligations linked to freedom, it also situates the social realities of the future, chronic condition, and health within a normative and prescriptive system still controlled by physicians and adults. To a lesser extent, physicians use discursive practices that frame the youth responsibility in terms of their decision-making agency, particularly through the use of the verb “to want” (23 occurrences), which focuses on the youth personal desires.^

“But it wouldn’t be a bad idea to … because right now, there’s babysitting, but perhaps there are centers … **if you want** to take on some side jobs with children, then within the framework of …”

“Well, **if you want**, but it’s not ideal; we’ll have trouble examining your knees and for the balance…”

(Juliette TPC, crohn's and hemorrhagic rectocolitis, 18 y.o.)

“**If you want**, and then we’ll (return) the thing like that. I’ll tell dad that you’ll be leaving quickly.”

(Kensia TPC, crohn's and Hemorrhagic rectocolitis, 17 y.o.)

^Secondly, physicians adopt a didactic discourse through explanatory practices using metaphors or concrete situations (in the form of exercises) in case of misunderstanding or to explore the youth reasoning abilities. This discursive practice places TCP within an educational and health promotion approach.^

“Physician: If you have a crisis this afternoon, what will you do?”

Manil: I have my parents.

Physician: But for example, if your parents don’t answer and you’re, I don’t know, on vacation in Spain with friends, and you’re there, what will you do?”

(Manil TPC, Spondyloarthropathy, 16 y.o.)

c. Positioning of the Interlocutors: What Social Reality Emerges from the Discourse

i. The youth as a “Whole Person”

^The analysis highlights an understanding of the youth in their entirety. The healthcare discourse explores their emotions, activities, wishes, and overall health, which are not solely related to the chronic condition. Physicians do not reduce the young person to their medical condition but view them as a complete individual:^

“Ibtissem: When I’m stressed, I have to listen to music. When I’m anxious, like last year when I was taking my diploma, well… I listened to music all the time… when I’m not feeling well… when I’m upset too…

Physician: Does it help you?

Ibtissem: Hmm!”»

(Ibtissem TPC, Type 1 diabetes, 16 y.o.)

ii. The youth in Transition as a Changing and Feeling Body

TPCs predominantly address chronic condition from the perspective of sensations and the body as a changing entity with new needs. The medical examination serves as a foundational moment for discourse on the body in transition to adulthood. The youth as a transitional being thus emerges through their “sensational” body, which experiences and feels emotions through subjective experiences and representations, often leading to practices of body dissatisfaction frequently associated with social comparison.

“[…] Physician: Do you wait to be in the water because… you don’t like being in front of others…?

Léa: Actually, it’s knowing that afterwards, everyone is looking… because often when we go to the pool, people like to watch and…

Physician: Hmm-hmm…

Léa: … it’s better in the water!

Physician: Is it because of scars, is it because of…

Léa: Yes, especially there…”

(Léa TPC, nephrotic syndrome, 18 y.o.)

iii. Health Prevention Practices

The discourse on health themes encourages the youth to change certain lifestyle habits or at least reflect on altering some behaviors, particularly regarding physical activity, screen time, sleep, and sexuality. Physicians, for example, discuss contraceptive methods, sexually transmitted diseases, and risky behaviors to raise awareness and inform youth about health-promoting behaviors they should adopt.

“Physician: You know that you can get pregnant from the very first sexual intercourse…

Abdoul: Hmm-hmm…

Physician: … you can also get diseases from the very first sexual intercourse, so you really need to protect yourself…

Abdoul: Hmm-hmm.

Physician: … use condoms, and then when you know each other better, there are other contraceptive method”

(Abdoul TPC, Allergic asthma, 17 y.o.)

iv. The youth in a Learning Situation for Self-Management

The physicians' discourse is structured by a combination of prescriptive statements and educational formulations aimed at fostering young person self-management. The youth is thus positioned in the role of a learner throughout the consultation, engaging in active learning and exploring their experiential knowledge. Self-management practices are explored and encouraged.

“Juliette: Well, I’m the one who does the injections (smile), so yes, I think about it, yes! (laughs)

Physician: Alright… so you’re the one who does it, you’re the one who goes to the pharmacy!?

Juliette: Oh yes, yes, I’m the one who handles reserving, calling…

Physician: Reserving the…

Juliette: … the medication…

Physician: Yes, yes, yes, okay! So you manage it, yes!? That’s right, and in terms of managing the illness technically and all, I still have the impression that you’re already very independent!”

(Juliette TPC, crohn's and hemorrhagic rectocolitis, 18 y.o.)

v. The Young Person as An Adult

At the end of the consultation, the young person is invited to reflect on their professional aspirations, future living situation, and how they will learn to manage their medical appointments independently:

Physician: Do you think about being an adult, having a wife, or…?

Ronan: No, not for now.

Physician: Not at all!?

Ronan: No

Physician: Do you see yourself in fifteen years as a father of a family or…?

Ronan: Yes, I would like that, yes…

Physician: And could the illness… make things more complicated or prevent you from meeting people?

Ronan: I don't think so.

(Ronan TPC, Hirschsprung's disease, 15 y.o.)

The questions about the future, especially regarding the transition to adult care, allow the youth to express their beliefs and representations and encourage their imagination through open-ended questions (“*How do you see it?; What do you imagine?; What does it represent?; How do you view your transition to adult care?*”). However, it is noteworthy that discussions about adulthood primarily occur at the end of the consultation, when family members (parents or siblings) are invited to join the discussion if they are present, creating a contrast between the discourse on adulthood and the context in which the youth is potentially regressed to a childlike role by the presence of their parents or siblings. This dominance in the corpus can be explained by the fact that at the end of TPC, the physician provides a summary to the youth of the main points discussed during the consultation. This summary is firmly oriented toward projecting into the future and adulthood.

## 4 Discussion

Our study aims to understand how a TPC shapes the social construction of adulthood for youth with CC. We conducted a critical discourse analysis using data collected in a natural context: recordings of 26 TPCs. To our knowledge, there is no research on how discourse is utilized during preparatory interventions for transitioning to adult care to bring forth a specific social reality of the transition.

Our results show that TPCs introduce the social reality of experiential health, based on the subjective experience of the chronic condition in terms of self-perceptions, future perceptions, and feelings.^31^ In contrast to other youth medical consultations, which focused solely on objective health indicators or medical criteria,^32^ the TPC functions as a phenomenological examination of the youth with CC, exploring health, life events and their projection into the future as they appear to the youth with CC consciousness.^33^ The process is phenomenological in that the youth with CC is perceived as a body-subject,^34^ becoming aware of their health condition and future possibilities through embodied experience. Our discourse analysis indicates that physicians focus on how the youth with CC makes sense of this embodied experience, particularly during the medical examination part of the TPC. The physicians' questioning explicitly explores the youth with CC feelings and the impacts of the chronic condition on their daily life, leading to the construction of a discourse on the experience of chronic condition during the transition to adulthood. This approach, which adolescents in medical consultations are asking for,^35^ also differs from the “transition readiness” logic, which focuses on the objective measurement of mental and behavioral readiness for transition ^36^ and pays little attention to the context and individual experience of the transition.^37^ The TPC thus opens a unique space-time in the youth with CC care pathway,^38^ offering a dialog on the experience of transitioning to adulthood with a chronic condition, addressing both the experiential (feelings and affects) and cognitive dimensions of the lived experience (meaning given) rather than only focusing on the sick body or normative behaviors expected.

While developmental psychology research highlights that identity development is a central concern for youth with CC ,^39^ focusing on the process of integrating the chronic condition into the self-concept, TPCs address identity only minimally in an explicit or cognitive manner. The experience of *being ill* is primarily understood through the dimension of lived experience, that is, the body that feels and also transforms (puberty), shifting the identity into a dynamic, evolving, "in transition" state. The implementation of TPCs by physicians, rather than by allied health professionals or psychologists, may be a hypothesis for the prominence of this bodily perspective. Indeed, this characteristic facilitates the clinical examination conducted during the TPC. Our study's analyses showed that this moment represents a particularly intense period of discourse on the lived experience and bodily transformations. The emerging social reality is less about a narrative on identity and possible selves and more about an ongoing bodily transition that informs the youth with CC about their health and self. Kelly et al. ^40^ highlight that the clinical examination is important because it allows clinicians to enter into a subjective relationship with patients. In this sense, TPCs fulfill the need to address the psychophysical challenges faced by youth with CC.^41^ The theoretical background and the roles of the professional leading TPC thus have a major influence on its content and the social construction of the transition process.

The cognitive dimension of the experience of transitioning to adulthood with a chronic condition is primarily addressed through the lens of autonomy and self-management. The discourse thus directs the reality of the transition towards knowledge and skills for self-care, as well as practices of responsibility. While educational statements and the supportive exploration of the youth with CC knowledge and practices structure the physicians’ discourse, the heavy emphasis on responsibility and health practices (“*you must*”) may paradoxically reflect the social expectation of autonomy (“*You are free… but you must*”). In this sense, TPCs align with patient education interventions aimed at developing skills for self-management through a pedagogical approach integrated into care.^42^ They embody the same virtues (the intention to empower the patient) and the same limitations (the risk of normalizing the patient towards adherence practices).^43^ In our study, the analysis of the verbatims shows that the empowerment of the youth with CC within TPCs mainly involves guiding them towards autonomy (“*You are free… so you must*”) in a preventive and educational approach, thus creating meanings about the notion of empowerment in terms of what it allows and what it entails. We can also note that the term " (invisible) disability" is never used in the TPCs studied. However, it is increasingly present in social discourse and could represent a social reality of illness that is even more focused on the obstacles encountered in growing up according to one's own goals.

Thus, the objective of TPCs might prove to be multiple: clinical examination, assessment of global health needs, or an educational/preventive approach. Discourse analysis shows that linguistic practices associated with all three fields are present. However, patients’ cognitive capacities (vigilance, processing, understanding, memorization) are described as often limited and call for clarification of educational moments versus other moments (consultation, assessment…) to support learning.^44^ Nevertheless, the complexity of TPC shows that these three practices are intertwined and interrelated (for example, the medical examination leads to exploring bodily sensations and transformations) and that their integration makes sense as a unique social reality.

As highlighted by Stokoe et al.,^24^ discourse analysis calls for a partnership with field teams to reflect on the practices engaged in and to propose avenues for improvement. In this perspective, the *AD’venir* team tend to adapt the TPC process to each youth’s needs, in particular its developmental stage and its family environment. Greater weight is done to the expertise of other healthcare professionals (psychologist, nurse) on the adolescent and their parents, complementary to medical approach.

## 5 Limitations

While our study provided a better understanding of the physician-youth with CC relationship in a natural context during a TPC, the perspective of the both actors was not explored. Therefore, we were unable to triangulate our results with information about the experiences of the youth with CC or physicians regarding the TPCs. Additionally, data collection stopped when the care team perceived a recurrence of themes and no longer identified new discussion points in the consultations. This stopping criterion was pragmatic and not based on a systematic saturation procedure. We could not select the recordings from a larger total quantity using a purposive sampling strategy that might have allowed us to study specific situations. in the context of this study, we considered that, although the various conditions encompass different clinical realities, they share common issues related to chronicity and the transition to adult care. The research therefore adopts a non-categorical approach to chronic conditions, acknowledging the existence of psychosocial invariants associated with chronicity. However, this approach limits attention to the clinical specificities of each condition. Furthermore, we did not examine the role and discourse of parents or siblings, who were present at the end of the consultation. Their contributions were limited, as they spoke only briefly at the conclusion of the TPC (approximately two minutes), and not on a systematic basis. For this reason, they were not included in the present analysis. Finally, the TPCs analyzed were conducted at the launch of the *AD’venir* platform. We can hypothesize that the team's practice, combined with supervision sessions and a better understanding of the system, has now altered both the content and the form of the discourse within the TPCs.

## 6 Conclusions

As interventions for healthcare transition aimed at empowering youth with CC become increasingly prevalent, it is essential to examine real-world contexts to gain a deeper understanding of the underlying processes. Our discourse analysis reveals how an innovative TPC constructs the social reality of transitioning to adulthood as a process involving a changing body, chronic condition management as an agentic process, and health as an experiential and bio-psycho-social phenomenon. The TPC functions as a social learning tool, offering youth with CC insights into themselves and their potential futures. We highlighted that transition programs involve language practices that situate the process within psychological and social realities, which are not neutral. The interactions between healthcare providers and youth constitute social learning situations, informing the youth about themselves and their potential. Future research should focus on analyzing real-world data to advance knowledge of the mechanisms and social learning processes specific to youth with CC. This perspective is crucial for the field of transition from pediatric to adult care, where educational practices are often insufficiently grounded in theoretical models of human development. Additionally, this study underscores the importance of understanding transition through the individual’s experiential reality, including the bodily sensations and expressions articulated by the youth with CC. This approach aligns with the patient experience framework, which is increasingly recognized, and should be further developed to document the psychosocial support needed from the healthcare system during the transition to adulthood.

## CRediT authorship contribution statement

**Mellerio Hélène:** Writing – review & editing, Writing – original draft, Validation, Conceptualization. **Paul Jacquin:** Writing – review & editing, Validation. **Enora Le Roux:** Writing – review & editing, Validation. **Agnes Dumas:** Writing – review & editing, Validation. **Clémentine Boudroit:** Validation, Methodology, Formal analysis. **Maxime Morsa:** Writing – review & editing, Writing – original draft, Methodology, Formal analysis.

## Consent to Participate

Participants were orally informed about the research, received an information letter and provided verbal consent (parents provided verbal consent when the participant was under 18).

## Ethical statement

Our study was approved by the Robert Debré Ethics Committee approved the protocol (IRB no. 2016/268).

## Funding

This research did not receive any specific grant from funding agencies in the public, commercial, or not-for-profit sectors.

## Declaration of Competing Interest

The authors declare that they have no known competing financial interests or personal relationships that could have appeared to influence the work reported in this paper.

## Data Availability

The data that has been used is confidential.
